# The effect of parity on obstetric and perinatal outcomes in pregnancies at the age of 40 and above: a retrospective study

**DOI:** 10.3325/cmj.2021.62.130

**Published:** 2021-04

**Authors:** Simten Genc, Cagdas N. Emeklioglu, Basak Cingillioglu, Erhan Akturk, H. Turhan Ozkan, Veli Mihmanlı

**Affiliations:** University of Health Science, Okmeydani Training and Research Hospital, Obstetrics and Gynecology Department, Istanbul, Turkey

## Abstract

**Aim:**

To examine the characteristics of pregnancies at a very advanced age and the effect of parity on adverse obstetric outcomes.

**Methods:**

We retrospectively reviewed the records of women who gave birth at the Obstetrics and Gynecology Department of Okmeydanı Training and Research Hospital between January 2012 and December 2019. Overall, 22 448 of women were younger than 40 and 593 were aged 40 and older. Women aged 40 and older were divided into the primiparous (52 or 8.77%) and multiparous group (541 or 91.23%).

**Results:**

Significantly more women aged 40 and older had a cesarean section. The most common indications for a secondary cesarean delivery in both age groups were a previous cesarean procedure or uterine operation. The most frequent indication for primary cesarean section in both groups was fetal distress. Cesarean section rates due to non-progressive labor, fetal distress, and preeclampsia were significantly more frequent in primiparous women compared with multiparous women aged 40 and older. In primiparous women, fetal birth weight was lower and preeclampsia/gestational hypertension frequency were higher.

**Conclusion:**

Since primiparity was a risk factor for lower fetal birth weight and preeclampsia/gestational hypertension in the age group of 40 years and above, more attention should be paid to the follow-up and treatment of these patients.

Due to social and economic problems, career priority, and prolonged education, an increasing number of women choose to give birth at an advanced age. Their choice is facilitated by the availability and efficacy of contraceptive methods and assisted reproductive technology (ART) (1). Still, the rate of nulliparity at advanced age increases, while the parity rate decreases (2).

The International Federation of Gynecology and Obstetrics (FIGO) uses the term “advanced maternal age” for pregnancies at the age of 35 and over, and the term “very advanced maternal age” for pregnancies at the age 40 and over. In these pregnancies, chronic diseases and medical problems are more common and these women constitute a high-risk patient group (3).

Most studies have shown that the advanced maternal age increases the risk of hypertension, gestational diabetes mellitus, postpartum hemorrhage, premature birth, cesarean procedure, intrauterine growth retardation, and perinatal mortality (4,5). However, studies comparing the outcomes in primiparous and multiparous women pregnant at an advanced maternal age are scarce, and the evidence for some of the outcomes is conflicting. Therefore, the aim of our study is to examine the characteristics of pregnancies at a very advanced maternal age and to assess the effect of parity on negative obstetric outcomes.

## MATERIALS AND METHODS

We retrospectively reviewed the birth records of 23 490 women who gave birth at 24 gestation weeks or more in the Obstetrics and Gynecology Department of the University of Health Science Turkey Okmeydanı Training and Research Hospital between January 2012 and December 2019. A total of 449 women were excluded because they were younger than 18. Among the remaining 23 041 women, 12 548 (ages below 40: 12297; ages 40 and over: 251) had normal spontaneous births and 10 493 (ages below 40: 10151, ages 40 and over: 342) had a cesarean delivery. Overall, 22 448 women were under the age of 40 and 593 were aged 40 or older. Women who were 40 and older were divided into the primiparous (52 or 8.77%) and multiparous group (541 or 91.23%).

The groups were compared in terms of the rate of gestational hypertension, preeclampsia, gestational diabetes mellitus (GDM), placental abruption, preterm labor, myoma uteri, placenta previa, maternal chronic diseases, multiple pregnancies, fetal macrosomia, birth weight, mode of delivery, gender, APGAR score, polyhydramnios, oligohydramnios, premature rupture of membranes (PROM), intrauterine fetal loss (fetal demise), and small for gestational age (SGA) fetus size.

GDM was diagnosed in patients at 24-28 gestation weeks with a two-step approach proposed by the American College of Obstetricians and Gynecologists. If the blood glucose level one hour after 50-g oral glucose tolerance test was 140 mg/dL (7.8 mmol/L) or higher, a 100-g glucose tolerance test was performed. The patients with two or more high values were diagnosed with GDM (6).

Preeclampsia and gestational hypertension was diagnosed according to the International Society for the Study of Hypertension in Pregnancy (7). Gestational hypertension was defined as systolic blood pressure of 140 mm Hg and above and/or diastolic pressure of 90 mm Hg and above for two measurements within 4 hours in a previously normotensive pregnant woman with no obvious proteinuria after the 20th gestation week. Preeclampsia was defined as the addition to these findings of 300 mg and more proteinuria in a 24-hour urine sample or 2 (+) proteinuria in urine for two times.

Macrosomia was defined as fetal weight equal or above 4000 g. Low birth weight was defined as fetal weight under 2500 g. Fetal loss (fetal demise) was defined as intrauterine fetal loss after 24 weeks of gestation. PROM was defined as a rupture of fetal membranes two hours before the labor onset. Small for gestational age (SGA) size was defined as the birth weight below the 10th percentile according to the gestational age.

Deliveries before the 37th gestation week were defined as premature and those after the 42nd gestation week were defined as post-mature.

The number of newborns with an APGAR score below 7 in the first and fifth minute was also noted.

Polyhydramnios was defined as the amount of amniotic fluid 8 cm above the single quadrant measurement or 20 cm above the total of four quadrants in ultrasonographic measurements. Oligohydramnios was defined as the amount of amniotic fluid 2 cm below the single quadrant measurement or 5 cm below the total of four quadrants.

This study was performed in line with the principles of the Declaration of Helsinki and was approved by the Ethics Committee of University of Health Sciences, Okmeydanı Training and Research Hospital (8670771-514.10).

### Statistical analysis

The normality of distribution was evaluated with the Kolmogorov-Smirnov test. Data are expressed as mean ± standard deviation, median (minimum-maximum), and count (percent) values where appropriate. Quantitative data were tested with the *t* test and Mann-Whitney U test. The Kruskal-Wallis test was used when there were more than two subgroups of ordinal data. Qualitative data were tested with the chi square, continuity (Yates), and Fisher exact test. *P* < 0.05 value was considered significant. Statistical analysis was performed with the SPSS, version 25 (IBM Corp., Armonk, NY, USA).

## RESULTS

The study enrolled 22 448 women between the ages of 18 and 40 (97.5%) and 593 women aged 40 or more (2.5%). Overall, 12 297 (54.8%) of 22 448 women younger than 40 had a spontaneous delivery and 10 151 (45.2%) had a cesarean procedure. In contrast, 251 (42.3%) of 593 women aged 40 or more had a spontaneous delivery and 342 (57.7%) had a cesarean delivery. Women who gave birth at a very advanced maternal age had a significantly higher frequency of cesarean sections (*P* < 0.001). The most common indication for a secondary cesarean delivery in both age groups was a previous cesarean procedure or uterine operation. The most common indication for a primary cesarean delivery in both age groups was fetal distress. The two age groups did not differ in the number of cesareans deliveries with the indications of progress failure, cephalopelvic disproportion, fetal distress, malpresentation, cordon prolapses, preeclampsia/gestational hypertension, multiple gestation, macrosomia, and placental disorders (Table 1).

**Table 1:** Indications for cesarean sections in women younger than 40 and aged 40 and older

	No. (%) of women		
	<40 y old (n = 10 151)	≥40 y old (n = 342)	P
Repeat cesarean section	5551 (54.7)	170 (49.7)	0.069
Progress failure	863 (8.5)	30 (8.8)	0.86
Cephalopelvic disproportion	376 (3.7)	12 (3.5)	0.851
Fetal distress	1467 (14.5)	51 (15)	0.812
Malpresentation*	578 (5.7)	27 (7.8)	0.086
Cordon prolapse	26 (0.3)	3 (0.9)	0.067
Preeclampsia/hypertension	462 (4.5)	23 (6.7)	0.06
Multiple gestation	216 (2.1)	5 (1.5)	0.399
Macrosomia	372 (3.6)	12 (3.5)	0.88
Placental disorders	240 (2.4)	9 (2.6)	0.743

*breech, shoulder, and transverse lie presentation.

While 1 patient (2.8%) in the primiparous group underwent a cesarean section due to a previous myomectomy, 169 patients (55.2%) in the multiparous group underwent a cesarean section due to a previous uterine operation or cesarean section. The number of cesarean sections due to non-progressive labor, fetal distress, and preeclampsia was significantly higher in primiparous women (*P* = 0.001, *P* = 0.003, *P* = 0.001, respectively). There was no significant difference between the groups in the number of cesarean sections due to cephalopelvic disproportion, presentation anomalies, multiple pregnancy, macrosomia, and placental anomalies (Table 2).

**Table 2:** Primary cesarean indications for women aged 40 years and older

	No. (%) of women		
	primiparous	multiparous	P
Progress failure	9 (25)	21 (6.9)	0.001
Cephalopelvic disproportion	2 (5.6)	10 (3.3)	0.366
Fetal distress	12 (33.3)	39 (12.7)	0.003
Malpresentation*	1 (2.8)	26 (8.5)	0.335
Cordon prolapse	-	3 (1)	na
Preeclampsia/hypertension	7 (19.4)	16 (5.2)	0.001
Multiple gestation	2 (5.6)	3 (1)	0.088
Macrosomia	1 (2.8)	11 (3.6)	1
Placental disorders	1 (2.8)	8 (2.6)	1

*breech, shoulder, and transverse lie presentation.

The median age of primiparous women was 40 years and that of multiparous women was 41 years. The average gravidity was 2 in primiparas and 4 in multiparas, and the difference was significant (*P* = 0.001). No significant anemia was detected in either group. Primiparas gave birth at 37.38 ± 2.8 gestation weeks, and multiparas at 37.88 ± 2.52 (*P* = 0.174). Newborns in the primiparous group had a lower birth weight (*P* = 0.019): 2927.69 ± 715.44 g vs 3158.21 ± 669.02 g in multiparas (Table 3).

**Table 3:** Comparison of primiparous and multiparous pregnancies in women aged 40 years and older

	Pregnancies		*P*
	primiparous	multiparous	
Age (years)*	40 (40-48)	41 (40-49)	0.498
Gravidity*	2 (1-4)	4 (2-14)	0.001
Abortion*	1 (0-3)	1 (0-6)	0.320
Hemoglobin†	11.43 ± 1.32	11.16 ± 1.37	0.175
Hematocrit†	34.31 ± 3.93	33.83 ± 3.76	0.384
Birth week†	37.38 ± 2.8	37.88 ± 2.52	0.174
Birth weight (gram)†	2927.69 ± 715.44	3158.21 ± 669.02	0.019
**Maternal diseases and comparison of maternal and fetal outcomes**	No. (%) of women		
Preeclampsia/gestational hypertension	8 (15.4)	29 (5.3)	0.010
Gestational diabetes mellitus	4 (7.7)	41 (7.5)	1
Type 2 diabetes mellitus	1 (1.9)	5 (0.9)	0.425
Uterine leiomyomas	2 (3.8)	7 (1.3)	0.183
Asthma	1 (1.9)	17 (3.1)	0.521
Epilepsy	0	1 (0.2)	na
History of thrombosis	2 (3.8)	3 (0.6)	0.064
Heart diseases	0	4 (0.7)	na
Thyroid diseases	1 (1.9)	25 (4.6)	0.719
**Comparison of maternal and fetal outcomes**			
Polyhydramnios	1 (1.9)	10 (1.8)	1
Oligohydramnios	5 (3.6)	13 (2.4)	0.597
Multiple pregnancy	2 (3.8)	3 (0.6)	0.064
Abruptio placentae	0	4 (0.7)	na
Premature rupture of membrane (PROM)	3 (5.8)	17 (3.1)	0.407
Placenta previa	1 (1.9)	7 (1.3)	0.522
Small for gestational age	4 (7.7)	19 (3.5)	0.134
Prematurity	14 (26.9)	147 (27)	1
Fetal demise	1 (1.9)	14 (2.6)	0.615
Assisted reproductive technology	3 (5,8)	0	na
Low birth weight (<2500 g)	11 (21)	71 (13)	0.109
Fetal macrosomia (>4000 g)	1 (1.9)	43 (8)	0.163
Apgar score <7 at 1 min	5 (10)	46 (9)	0.795
Apgar score <7 at 5 min	0	0	na
Cesarean delivery	36 (69.2)	306 (56.6)	
Vaginal delivery	16 (30.8)	235 (43.4)	0.105
Male fetus	28 (53.8)	292 (53.7)	
Female fetus	24 (46.2)	249 (45.8)	0.980

*median (minimum-maximum).

†data presented as mean ± standard deviation.

The most common maternal disease in the primiparous group was preeclampsia/gestational hypertension (15.4%), while in the multiparous group it was GDM (7.5%). Among all maternal diseases, only preeclampsia/gestational hypertension was significantly more frequent in primiparas (*P* = 0.01). No significant difference was found in the frequency of gestational diabetes, type 2 diabetes, myoma uteri, asthma, epilepsy, thrombosis history, maternal heart diseases, and thyroid diseases.

While placental abruption was not encountered in primiparous patients, it was found in 0.7% of the multiparous group. The use of ART was very rare in our study – it was recorded in 3 women (5.8%) in the primiparous group and no women in the multiparous group (Table 3).

The multiparous group showed no significant decrease in the frequency of PROM, placenta previa, SGA fetuses, prematurity, polyhydramnios, oligohydramnios, fetal demise, newborns with low birth weight (<2500 g), macrosomia (>4000 g), and first-minute APGAR score. There was also no significant difference in fetal sex and birth patterns between the groups.

## DISCUSSION

This study, conducted on the largest sample so far, showed that the primiparous group aged 40 and older had a higher risk of preeclampsia and lower fetal weight compared with the multiparous group of the same age. Maternal characteristics and obstetric results should be considered in detail and should be monitored carefully, especially in nulliparous women with advanced maternal age pregnancies.

Women with advanced maternal age pregnancies have a significantly increased risk of cesarean deliveries (8). However, the factors associated with this increased risk are not fully understood (9,10). The rates of cesarean deliveries for women older than 40 years range from 20% to 58% (11-13). In the present study, this rate was 57.7%. The high rate can be explained by the fact that our hospital as a referral center treats more difficult cases transferred from other hospitals, and by a high risk of maternal chronic diseases, fetal indications, and obstetric complications in women over the age of 40.

In our study, the rate of cesarean deliveries was high in both primiparous and multiparous advanced maternal age pregnancies. Literature reports attribute the higher rate of cesarean deliveries in women over 40 years to the “precious baby syndrome” (14). Primiparous women at advanced maternal age who used ART are expected to have higher cesarean rates (15). However, these patients were rare in our sample. Therefore, cesarean section rates were not compared between primiparous and multiparous groups who used ART.

Our study found no significant difference between the primiparous and multiparous group in the frequency of premature births. Spontaneous preterm delivery and iatrogenic preterm delivery are caused by different pathophysiological mechanisms. Older primiparas may more frequently have iatrogenic premature delivery. Alshami et al (16) and Jolly et al (17) reported that preterm delivery risk did not increase due to the increase in maternal age only, but due to additional conditions, such as hypertension and bleeding (16,17). However, both of these studies have compared pregnancies in advanced maternal age to young pregnancies without distinguishing between primiparous and multiparous women. Chan and Lao divided pregnant women over 40 into the nulliparous and multiparous group but compared them with younger women (18). They found a higher frequency of preterm deliveries in nulliparas and multiparas in the advanced maternal age group, and observed that preterm birth before 37 weeks was independently associated with advanced maternal age, irrespective of parity. Ngowa et al (19) found an increase in preterm births in multiparous women. Contrary to other studies, our study compared primiparous and multiparous pregnant women in the advanced maternal age group.

The risk of gestational hypertension and preeclampsia increases with advanced maternal age (20,21). Duckitt et al found a 2-fold increase in the risk of preeclampsia in women aged 40 or older (22). In our study, the frequency of preeclampsia/gestational hypertension was significantly higher in primiparas. Our results are compatible with those from other literature reports (19,23,24). However, Chan and Lao found no significant difference between the two groups (18).

While some studies showed a relationship between low birth weight and nulliparity (13,18), others showed a relationship between low birth weight and multiparity (19). According to our study, even though fetal weight was significantly lower in the primiparous group, the two groups did not significantly differ when low birth weight (<2500 g) was examined. Newborns weighing <2500g were observed in 21% of primiparous women and in 13% of multiparous women.

Since pancreatic insulin sensitivity decreases with age, GDM is more common in women 40 years old or older (25). Although some studies reported that GDM frequency increased in advanced maternal age multiparous pregnant women (15), our study found no significant difference between primiparas and multiparas.

Placenta previa was reported to be more common in advanced maternal age pregnancies (15,26). Our study found no significant difference between primiparas and multiparas in the frequency of placenta previa, in accordance with the results by Kalayci et al (23).

Fetal loss is reported to be more common in advanced maternal age multiparous women (19,27), but some studies show it to be more common in primiparas (23). We showed no significant difference between primiparas and multiparas in this outcome. A meta-analysis of Jacobsson et al (28), analyzing a million and a half births, showed that the risk of perinatal mortality, intrauterine fetal death, and neonatal death increased with age. Yet, no link with parity was demonstrated (28).

While we did not observe any case of placental abruption in the primiparous group, we observed it in 4 patients in the multiparous group (0.7%). Some studies reported it to be more frequent in nulliparas (27), some studies reported that it was more frequent in multiparas (13), and some observed no difference (23).

Our study showed no significant difference between the first- and fifth-minute APGAR scores below 7 in the primiparous and multiparous groups, which is in accordance with the literature (29).

Other studies showed that fetuses with SGA were more common in older pregnant women (24,26). In our study, no difference according to parity was found. However, Kalaycı et al reported this condition to be more common in nulliparas (23).

The study limitations include the retrospective design and a lack of data on patients' race and educational status.

Pregnancies at a very advanced maternal age are likely to lead to perinatal and maternal complications and primiparity is a significant risk factor in this age group, so these patients should be carefully monitored in order to avoid maternal and fetal adverse obstetric outcomes.
